# Differential Regulation of PKM2, AMPK, and mTOR in Response to Insulin and Dietary Management

**DOI:** 10.3390/cells14060416

**Published:** 2025-03-12

**Authors:** Emily Broberg, Jillise English, Derek M. Clarke, Marley J. Shin, Benjamin T. Bikman, Paul R. Reynolds, Juan A. Arroyo

**Affiliations:** Department of Cell Biology and Physiology, Brigham Young University, Provo, UT 84602, USAjillenglish-234@byu.edu (J.E.); mshin-8765@byu.edu (M.J.S.);

**Keywords:** GDM, placental metabolism, PKM2, AMPK, mTOR

## Abstract

Gestational diabetes mellitus (GDM) affects placental metabolism, influencing both maternal and fetal outcomes. This study investigated the expression of metabolic regulators—Pyruvate Kinase M2 (PKM2), AMP-activated protein kinase (AMPK), and mTOR pathway components—in placental tissues from GDM pregnancies managed with either insulin (GDM-I) or dietary interventions (GDM-D). We hypothesize that metabolic adaptation in GDM is differentially regulated by treatment modality. This study analyzed 30 cases, including 10 control pregnancies,10 GDM-D cases, and 10 GDM-I cases. Analytical methods included immunofluorescence and immunoblotting. We observed an upregulation of PKM2 in both GDM-I and GDM-D placentas, suggesting enhanced glycolytic adaptation under GDM-induced metabolic stress. AMPK expression was significantly elevated in GDM-I and moderately increased in GDM-D placentas, potentially compensating for insulin resistance by promoting glucose uptake and energy homeostasis. Furthermore, mTOR pathway activation differed by treatment type, suggesting a treatment-specific mTOR response. The metabolic changes observed suggest that treatment modality in GDM may have direct implications for maternal and fetal health. Our findings indicate that while insulin and dietary management support metabolic adaptation in GDM, they do so through distinct mechanisms. These findings support a personalized approach in GDM treatment, where patient-specific metabolic responses should guide therapeutic decisions.

## 1. Introduction

Proper placental metabolism is essential for a successful pregnancy. The placenta is the primary site of nutrient and gas exchange between the mother and the fetus. Trophoblast cells within the placenta mediate energy-intensive processes such as nutrient transfer, hormone synthesis, and molecule secretion [1]. Placental nutrient metabolism is closely correlated with oxygen availability, as the placenta accounts for almost 40% of uteroplacental oxygen uptake [2]. Changes in nutrient supply to the fetus can affect its growth and development, potentially leading to obstetric complications [2].

Previous reports have established that tissues can undergo metabolic adaptations to meet ATP demands, often by promoting nonoxidative glycolysis under limited nutrient availability [1]. Such adaptations are significant in situations characterized by altered nutrient supply to the fetus, including gestational diabetes mellitus (GDM). This suggests that altered placental metabolism is essential in placental development and the onset of obstetric complications, such as those observed in GDM.

GDM is a common obstetric complication, affecting nearly 20% of pregnancies. This disease is characterized by insulin resistance and can lead to the development of hyperglycemia [3,4]. GDM pregnancies increase the risk of gestational hypertension, pre-eclampsia, and long-term health risks, including cardiovascular disease, obesity, and impaired carbohydrate metabolism, which may predispose both the mother and the infant to type 2 diabetes (T2DM) [5]. Although the mechanisms by which GDM contributes to an increased risk of metabolic disorders in both pregnant women and their offspring are not fully understood, aberrant placental glucose metabolism has been implicated [6].

Pyruvate kinase M2 (PKM2) is an enzyme in the glycolytic pathway not well characterized in the GDM placenta. This enzyme catalyzes a late step in glycolysis, transferring a phosphate group from phosphoenolpyruvate (PEP) to ADP. PKM2 is primarily expressed in cells with high nucleotide synthesis demands, such as proliferating cancer cells and developing embryonic [7,8]. PKM2 enables cells to adapt to different physiological states by forming highly active tetramers or low-activity dimers [9,10,11]. The switch between dimeric and tetrameric forms of PKM2 is essential for balancing energy supply and cell proliferation [12,13,14,15]. While the active PKM2 tetramer regulates glycolysis, dimeric PKM2 enters the nucleus to influence gene expression [12,13,14]. When glucose is abundant, PKM2 is activated, leading to glycolytic flux. When glucose is limited, and PKM2 is inactivated, glycolytic intermediates accumulate and are diverted into biosynthetic pathways [10,16]. The phosphorylation modification of PKM2 can increase its nuclear localization, thus favoring the dimeric form of this enzyme [17]. While much has been learned about PKM2’s role in cancer metabolism, it remains underexplored during normal placentation and in GDM-affected placentas.

PKM2 and AMP-activated protein kinase (AMPK) are critical regulators of cellular energy metabolism, often acting in response to metabolic stress and cellular energy requirements. AMPK is activated under low energy conditions (e.g., high AMP/ATP ratio), promoting catabolic processes to restore cellular ATP levels [18]. Activated AMPK is known to modulate metabolic pathways within cells and is regarded as a potential target for treating metabolic diseases such as diabetes [19,20]. Activated AMPK stimulates glucose uptake in various tissues, increasing insulin-mediated skeletal muscle glucose uptake in rodent diabetes models [6]. During GDM, AMPK activity is often reduced, impairing insulin signaling and increased glucose production, exacerbating hyperglycemia [21,22].

While previous studies have explored the roles of pyruvate kinase M2 (PKM2), AMP-activated protein kinase (AMPK), and the mechanistic target of rapamycin (mTOR) in various contexts, our study uniquely investigates their interplay within the placental tissue of pregnancies complicated by gestational diabetes mellitus (GDM). This approach addresses a critical gap in understanding how these metabolic regulators collectively influence placental function and fetal development in GDM settings. Notably, our research examines the differential phosphorylation patterns of Akt and AMPK and their subsequent regulation of mTOR activity in the placentas of GDM pregnancies, providing insights distinct from studies focusing on these pathways in isolation or non-gestational tissues [23].

The selection of PKM2, AMPK, and mTOR as focal points in our study is deliberate, given their pivotal roles in cellular energy homeostasis and metabolic signaling. PKM2 is integral to glycolytic flux and energy production, which are often dysregulated in GDM [24]. AMPK is a cellular energy sensor that activates pathways that restore energy balance under metabolic stress [25]. mTOR functions as a nutrient sensor, regulating cell growth and protein synthesis in response to nutrient availability [26]. Focusing on these regulators allows us to dissect the specific metabolic alterations occurring in the GDM placenta, which may differ from other metabolic pathways previously studied in this context. This targeted approach enables a more precise understanding of the mechanisms underlying placental dysfunction in GDM.

Thus, the aim of this study was two-fold: first, to investigate PKM2 expression in GDM, specifically in GDM patients treated with insulin (GDM-I) vs. dietary interventions (GDM-D); and second, to establish the correlation between AMPK, PKM2, and mTOR placental expression in GDM-I and GDM-D patients. To provide a more precise comparison, we directly analyzed differences between GDM-I and GDM-D rather than just comparing each group to controls.

Our findings suggest that metabolic dysregulation in the placenta, characterized by alterations in PKM2, AMPK, and mTOR signaling, may have significant long-term implications for maternal and fetal health. Such dysregulation can contribute to fetal overgrowth, increasing the risk of birth injuries and neonatal complications [23]. Furthermore, these placental metabolic alterations may predispose offspring to metabolic disorders, including obesity and type 2 diabetes, later in life [27]. Understanding these pathways provides critical insights into the developmental origins of health and disease, emphasizing the need for targeted interventions to improve placental function and reduce long-term metabolic risks for the offspring.

## 2. Materials and Methods

### 2.1. Human Placentas

Placental biopsies and paraffin-embedded tissues from GDM-I (gestational diabetes mellitus treated with insulin; n = 10), GDM-D (gestational diabetes mellitus treated with diet; n = 10), and term controls (non-GDM healthy pregnancies; n = 10) were obtained from the Research Center for Women’s and Infant’s Health Biobank, Toronto, ON, Canada. Gestational diabetes mellitus (GDM) was diagnosed based on a 75 g oral glucose tolerance test (OGTT), and samples were collected from placentas delivered either by C-section or vaginally.

Insulin treatment for GDM-I patients varied based on glucose levels and insulin resistance severity, with 10–50 units/day dosages. Diet control for GDM-D patients consisted of a low-glycemic-index meal plan focusing on carbohydrate restriction and portion control. No combined treatment (insulin and diet) samples were included in these studies.

The sample size of n = 10 per group was determined based on previous studies analyzing placental metabolic adaptations in GDM pregnancies [12,28].

To ensure valid comparisons, baseline characteristics such as maternal age, gestational age, and fetal birth weight were compared between GDM-I and GDM-D groups. No significant differences were observed between groups for these parameters (*p* > 0.05), confirming comparability between treatment groups. This minimizes the risk of confounding by gestational age or maternal metabolic status at baseline.

To ensure valid comparisons, baseline characteristics such as maternal age, gestational age, and fetal birth weight were compared between GDM-I and GDM-D groups. No significant differences were observed between groups for these parameters (*p* > 0.05), confirming comparability between treatment groups.

Table 1 shows the demographic information of the collected samples.

### 2.2. Immunofluorescence (IF)

IF was performed on paraffin-embedded placental sections as previously conducted in our laboratory [29]. Briefly, serial sections were incubated overnight with rabbit polyclonal antibodies against PKM2, activate or phospho (p) mTOR, pp70, and p4EBP1 (all from Cell Signaling, Danvers, MA, USA). Trophoblast layers were identified based on cytokeratin-7 staining (Dako, Carpinteria, CA, USA), a widely used marker for trophoblast differentiation. β-actin was chosen as a housekeeping protein for Western blot normalization due to its consistent expression in placental tissue across physiological and pathological conditions. To ensure accuracy, automated and manual quantifications were performed using the ImageJ software (Version 1.54p) and blinded analyses by two independent investigators. Anti-mouse fluorescein or Texas red-conjugated secondary antibody was incubated for one hour; 4′,6-diamidino-2-phenylindole dihydrochloride (DAPI) was used for nuclear counterstaining. Slides were viewed on a BX61 fluorescent microscope (20×) using the appropriate excitation and emission filters (fluorescein or rhodamine filters), with scale bars set at 100 μm.

### 2.3. Immunoblotting

Western blot analysis was used to determine the expression levels of PKM2, phospho (p) PKM2, pERK, and pAMPK in control, GDM-D, and GDM-I placental lysates, as well as in lysates of control and treated trophoblast cells (n = 10) [12]. Cell lysates (50 μg) were separated on a 10% SDS-PAGE gel and transferred onto nitrocellulose membranes. The membranes were blocked and incubated overnight with antibodies for PKM2, pPKM2, pERK, or pAMPK (all from Cell Signaling Technology, Danvers, MA, USA) or β-actin (Santa Cruz Biotechnology, Dallas, TX, USA). Membranes were then incubated with secondary IRDye antibodies (680RD donkey anti-goat and 680RD donkey anti-rabbit; LICOR, Lincoln, NE, USA) at room temperature for an hour. Membranes were developed on a Li-COR Odyssey CLx, and all results were normalized to β-actin loading control. Fluorescence densities were compared to control groups.

### 2.4. Statistical Analysis

Differences in PKM2, pPKM2, pERK, or pAMPK were determined between control and treated tissues (GDM-I and GDM-D) and between the fold changes between GDM-I and GDM-I using the Mann–Whitney test. Data are shown as mean  ±  SE. Significant differences between groups were noted at *p* < 0.05. Statistical analysis was performed with the GraphPad Prism 8.0 software.

## 3. Results

The placenta responds to metabolic stress by altering key signaling pathways, including mTOR, PKM2, and AMPK. Our findings demonstrate that the choice of GDM treatment influences these metabolic adaptations. Beyond molecular and metabolic changes, gestational diabetes mellitus (GDM) is associated with significant alterations in the placenta’s gross morphology and microscopic structure. Recent studies have identified notable differences in placental weight, villous structure, and vascularization in GDM pregnancies, which can contribute to altered placental function and fetal development [30]. GDM placentas tend to be larger, with increased placental weight and thickness, which may reflect compensatory mechanisms to maintain adequate nutrient exchange in response to hyperglycemia. However, these changes can also predispose the placenta to dysfunction, leading to adverse pregnancy outcomes such as fetal overgrowth and macrosomia.

Microscopically, GDM placentas often exhibit increased syncytial knots, fibrinoid necrosis, and villous immaturity, indicative of placental stress and maladaptive remodeling. These changes can impair nutrient transport efficiency and fetal oxygenation, potentially exacerbating metabolic dysregulation in the developing fetus—additionally, maternal hypertension—a common comorbidity in GDM—further compounds these placental abnormalities. The interplay between GDM and hypertension may increase oxidative stress and inflammation within the placenta, disrupting normal trophoblast function.

These structural changes align with the metabolic findings observed in this study, particularly the differential activation of mTOR, PKM2, and AMPK in GDM placentas. The increased trophoblast proliferation seen in GDM-I placentas, likely driven by mTOR activation, corresponds with observed increases in placental size and weight. However, excessive mTOR activation may also lead to trophoblast hypertrophy and impaired placental efficiency, negatively impacting fetal growth regulation. Similarly, AMPK activation in GDM-D placentas may reflect an adaptive response to maintain energy homeostasis despite these structural abnormalities.

Given these findings, future studies should integrate metabolic and histological analyses to fully understand the impact of GDM and hypertension on placental function. Assessing gross placental changes alongside molecular profiling could enhance the ability to predict pregnancy complications and guide treatment strategies that mitigate adverse placental adaptations.

### 3.1. mTOR Pathway Activation in GDM Placentas

**Figure 1:**
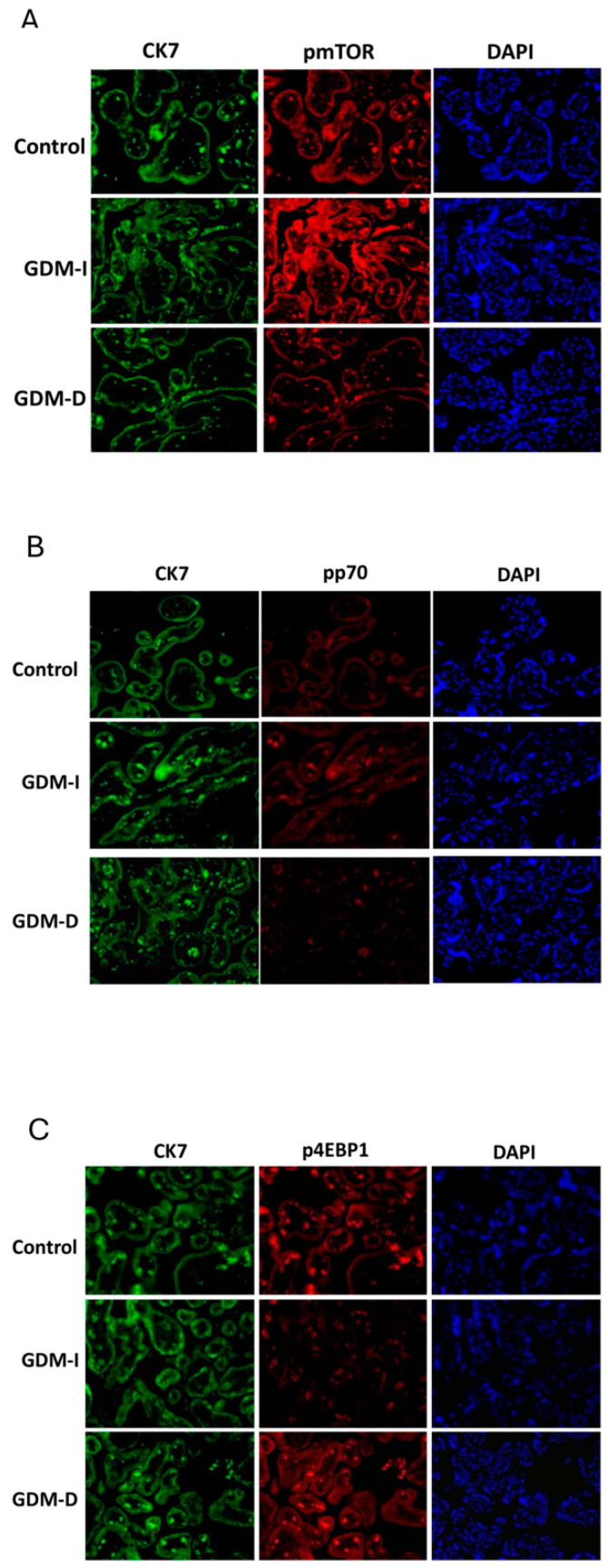
Placental mTOR pathway during GDM-I and GDM-D. Placental pmTOR (**A**), pp70 (**B**), and p4EBP1staining were performed between the control and disease placenta. Increased p-mTOR expression was only observed in the GDM-I placenta (**A**). pp70 was decreased in the GDM-D with no differences for the GDM-I (**B**). p4EBP1 was increased in GDM-D and decreased in GDM-I placentas compared to controls (**C**). Slides were viewed on a BX61 fluorescent microscope (20×) using the appropriate excitation and emission filters (fluorescein or rhodamine filters).

The mTOR pathway regulates metabolism in response to nutrient availability [12]. Increased mTOR activity is associated with the development of GDM during pregnancy [12]. Compared with controls, we observed an increased expression of active mTOR in the trophoblast cells of GDM-I placentas, while no changes were detected in the GDM-D tissues (Figure 1A). Importantly, direct comparisons between GDM-I and GDM-D revealed that GDM-I placentas exhibited significantly higher levels of active mTOR than GDM-D placentas (*p* < 0.05), suggesting insulin treatment may amplify mTOR signaling more than dietary management.

Active p70 did not show staining differences between controls and GDM-I patients (Figure 1B). In contrast, GDM-D placentas showed a decreased expression of active p70 compared with controls (Figure 1B). This decrease was also present when comparing active p70 between GDM-I and GDM-D placentas independent of control. Activated 4EBP1 expression was increased in GDM-D and decreased in GDM-I placentas compared with controls (Figure 1C). When comparing GDM-I with GDM-D placental samples, a significant increase in activated 4EBP1 was detected in the placenta of patients treated with diet. These findings suggest that insulin-treated GDM enhances mTOR activity, potentially affecting fetal growth, while dietary management results in a more restrained mTOR response. 

### 3.2. PKM2 Expression and Its Role in Glycolytic Adaptation

**Figure 2:**
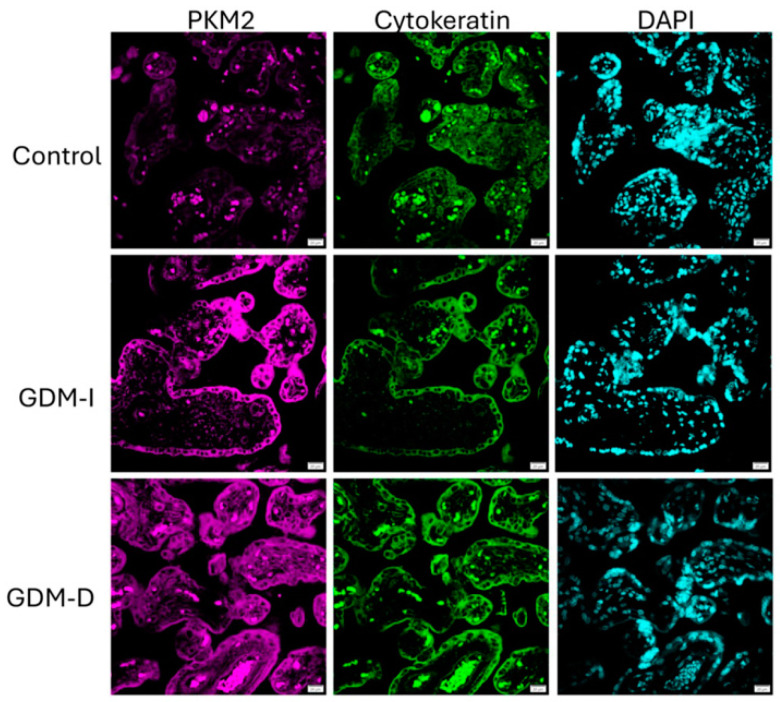
PKM2 expression and localization in the GDM-I and GDM-D placentas. The GDM-I placentas showed a higher syncytiotrophoblast expression of PKM2 than the GDM-D placentas. Slides were viewed on a BX61 fluorescent microscope (20×) using the appropriate excitation and emission filters with scale bars set at 100 μm.

**Figure 3:**
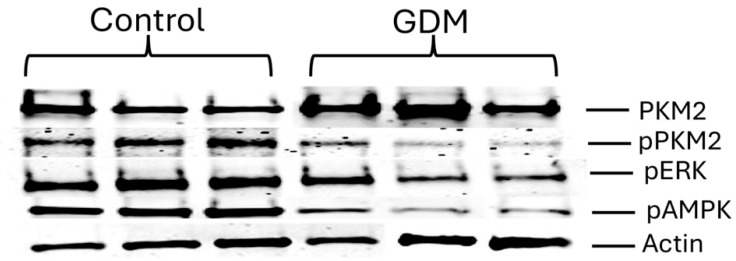
Characteristic western blot of proteins studied.

**Figure 4:**
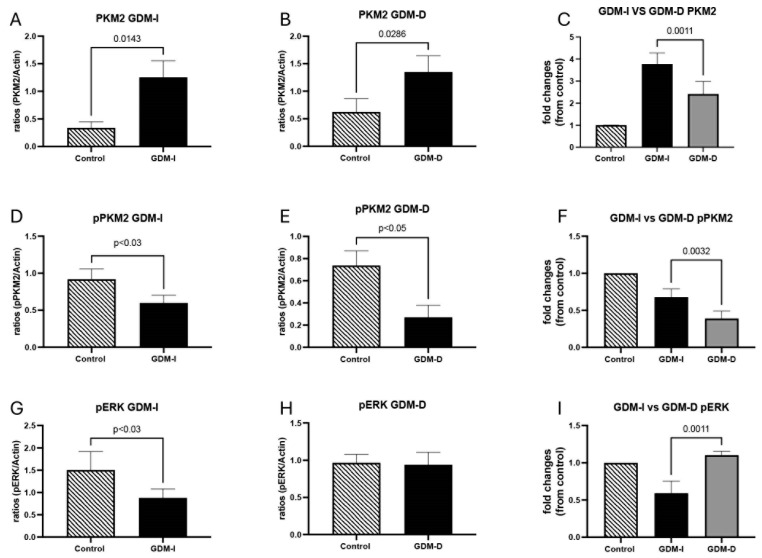
PKM2, pPKM2, and pERK protein in the GDM-I and GDM-D placentas. Immunoblot showed an increase in PKM2 in GDM-I and GDM-D compared to controls (**A**,**B**). A higher PKM2 fold change was observed in the GDM-I placentas compared to the GDM-D tissues (**C**). Placental pPKM2 was decreased in the GDM-I and GDM-D tissues compared to controls (**D**,**E**). The decrease in the GDM-D placental tissues was more pronounced than in the GDM-I tissue (**F**). pERK was decreased in the GDM-I placentas (**G**), while no differences were observed for pERK in the GDM-D placentas (**H**). GDM-D pERK expression is higher than in the GDM-I placental tissues (**I**). Data are shown with *p* ≤ 0.05 when compared to controls.

Immunofluorescence showed increased PKM2 expression in both GDM-I and GDM-D placentas compared with controls (Figure 2). When comparing treatments, treated placentas showed increased PKM2 expression localized primarily to the syncytiotrophoblast layer, highlighting its role in facilitating glycolytic energy production under hyperglycemic conditions (Figure 2). This increase in PKM2 was more pronounced in the diet-treated placenta than in insulin-treated patients. Immunoblotting was used to confirm protein expression in the placenta. Representative immunoblots of all the proteins studied are shown in Figure 3. PKM2 expression showed a 3.7-fold increase in the GDM-I placentas (median and IQR values of 1.38 and 0.48; *p* < 0.01) and a 2.0-fold increase in the GDM-D placentas (median and IQR values of 0.38 and 0.2; *p* < 0.04) as compared with controls (Figure 4A,B). When comparing PKM2 protein expression in the treatment samples, there was a 1.5-fold increase difference (*p* < 0.002) between the GDM-I placental tissues and the GDM-D ones (median and IQR values for GDM-I and GDMD groups were 11.1, 0.91 and 3.1, 0.33 and 0.48, respectively; Figure 4C). Phospho (p) PKM2, associated with the inactive form, was decreased 1.5-fold in GDM-I placentas (median and IQR values of 0.97 and 0.5; *p* < 0.03) and 2.7-fold (median and IQR values of 0.6 and 0.2; *p* < 0.05) in the GDM-D placentas as compared with controls (Figure 4D,E). Between samples, GDM-I showed a 1.7-fold higher expression of pPKM2 (*p* < 0.004) than the GDM-D placentas (median and IQR values for GDM-I and GDMD groups were 0.71 and 0.91 and 0.41, 0.33, and 0.93, respectively; Figure 4F). Previous studies have demonstrated that ERK activation can phosphorylate PKM2 [25]. Phosphorylated ERK (pERK) was decreased 1.7-fold in the GDM-I placentas (median and IQR values of 0.97 and 0.76; *p* < 0.03), while pERK levels did not change in the GDM-D placentas as compared with controls (median and IQR values of 0.97 and 0.33; *p* < 0.002; Figure 4G,H). Between samples, GDM-D showed a 1.9-fold higher expression of pERK (median and IQR values for GDM-I and GDMD groups were 0.55, 0.2, and 0.41 and 1.1 and 0.04, respectively; *p* < 0.002) than the GDM-D placentas (Figure 4I).

**Figure 5:**
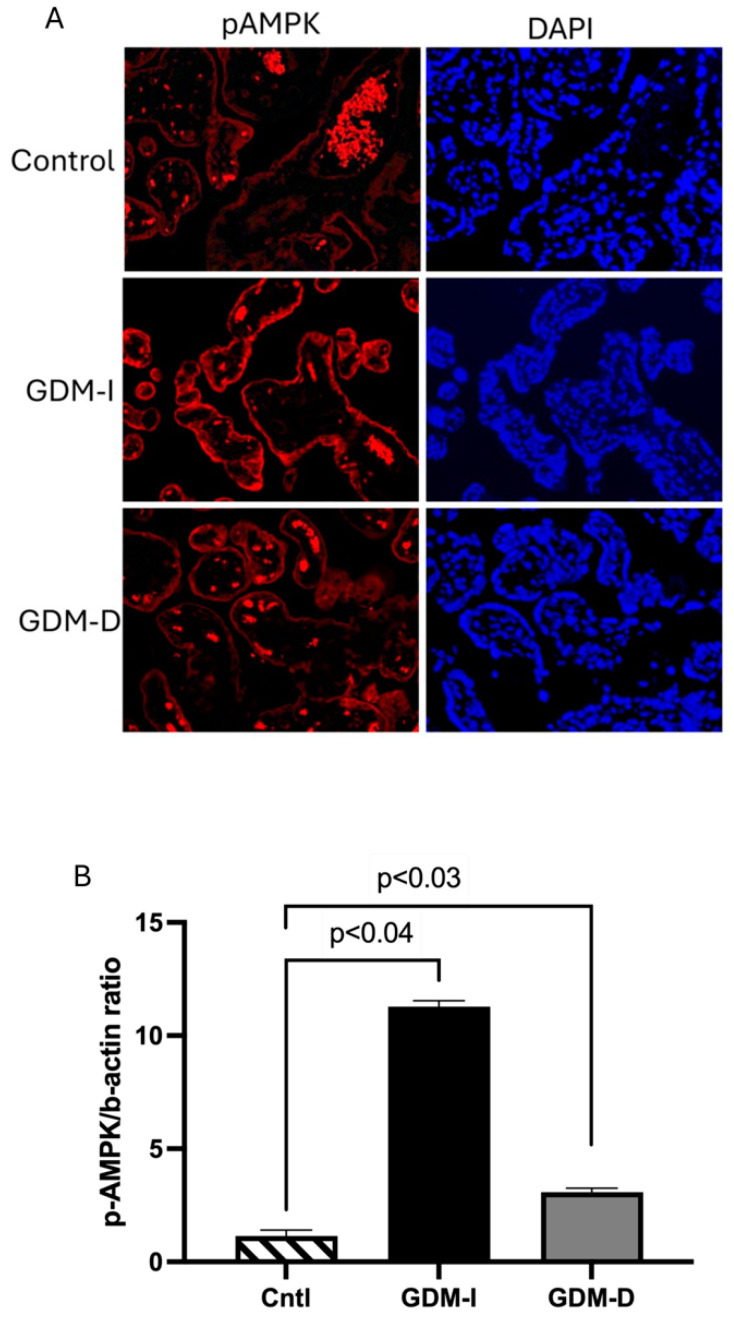
Activated (p) placental AMPK in GDM-I and GDM-D patients. Immunoblot was performed for pAMPK protein determination. pAMPK protein was activated in the syncytiotrophoblast of the GDM-I placentas, and no differences were observed in the GDM-D placentas (**A**,**B**). Data are shown with significance of *p* ≤ 0.05 when compared to controls. Slides were viewed on a BX61 fluorescent microscope (20×) using the appropriate excitation and emission filters (fluorescein or rhodamine filters).

AMPK, which promotes glucose metabolism, was increased in GDM placentas. Furthermore, PKM2 activation has been associated with AMPK activation in cancer cells [31]. pAMPK staining showed syncytiotrophoblast localization in both GDM-I and GDM-D placentas compared with controls (Figure 5A). Compared with controls, a higher increase in pAMPK expression was observed in the GDM-I than in the GDM-D placentas (Figure 5A). Immunoblotting confirmed the increased pAMPK expression, showing a 9.8-fold increase in GDM-I and a 2.7-fold increase in GDM-D compared with controls (*p* < 0.04 and *p* < 0.03, respectively) (Figure 5B). Statistical comparison between GDM-I and GDM-D demonstrated that PKM2 expression was significantly higher in the GDM-I placentas than in GDM-D (*p* < 0.05). Similarly, phosphorylated AMPK (pAMPK) expression was markedly increased in the GDM-I placentas compared to the GDM-D placentas (median and IQR values for GDM-I and GDMD groups were 11.2, 0.5, and 3.1, 0.3 respectively; *p* < 0.05), reinforcing the notion that insulin treatment induces a stronger metabolic response. 

## 4. Discussion

Gestational diabetes mellitus (GDM) is a prevalent pregnancy complication, with significant implications for both maternal and fetal health. GDM is associated with altered placental metabolism, which may contribute to adverse pregnancy outcomes. Often this complication manifests as hyperglycemia due to insulin resistance, which is associated with various adverse outcomes such as an increased risk of hypertensive disorders and long-term metabolic conditions for both mother and child [3,4]. Our study highlights critical insights into the molecular alterations within the placenta associated with GDM, focusing on pyruvate kinase M2 (PKM2), AMP-activated protein kinase (AMPK), and the mechanistic target of rapamycin (mTOR) pathway. A key observation was that the GDM-I and GDM-D placentas exhibited different metabolic adaptations. While PKM2 and pAMPK levels increased in both groups, the upregulation was significantly higher in the GDM-I placentas. Similarly, mTOR activation was enhanced in the GDM-I placentas compared to GDM-D, suggesting that insulin therapy may further exacerbate metabolic shifts compared to dietary management. It is important to note that the difference in metabolic response may be due to the severity of insulin resistance in GDM-I patients, which necessitates insulin therapy. This underscores the need to account for baseline metabolic differences between GDM-I and GDM-D when interpreting results. While both GDM-I and GDM-D placentas exhibited metabolic adaptations, the responses were distinct. A key question arising from these findings is whether the metabolic alterations induced by insulin treatment and dietary management operate through qualitatively different pathways or converge on similar metabolic mechanisms. Our data suggest that while both treatment modalities promote metabolic adaptation in the placenta, they do so via distinct regulatory influences. Insulin therapy in GDM-I enhances glucose uptake, which in turn amplifies PKM2 expression and mTOR activation, likely through the direct stimulation of the insulin-Akt-mTOR axis [32]. In contrast, dietary management in GDM-D appears to promote a more balanced metabolic state, with a less pronounced activation of mTOR but still enhanced glycolysis and AMPK upregulation, possibly reflecting an adaptive response to nutrient restriction rather than direct insulin signaling [4]. Despite these differences, both treatments ultimately modulate placental energy metabolism, suggesting that metabolic adaptation in GDM placentas may converge on common pathways but with distinct regulatory inputs depending on treatment strategy. Future studies should explore whether a combination of dietary and pharmacological interventions could optimize metabolic balance while mitigating excessive mTOR activation.

Although this study identifies significant metabolic differences in GDM-I and GDM-D placentas, these adaptations’ specific mechanisms remain incompletely understood. Our data indicate that PKM2, AMPK, and mTOR pathways are differentially regulated in response to insulin treatment versus dietary management. However, determining causality requires mechanistic studies using controlled in vitro models. Trophoblast cell cultures exposed to insulin and hyperglycemic conditions could provide direct evidence of insulin-mediated PKM2 and mTOR activation. Prior studies have shown that insulin signaling can modulate glycolytic enzyme expression through Akt-dependent pathways, suggesting that trophoblast culture models could be valuable in dissecting these regulatory mechanisms [33]. Additionally, the knockdown or pharmacological inhibition of PKM2, AMPK, or mTOR in trophoblast models could clarify whether these pathways are essential mediators of placental metabolic adaptation in GDM. Future research employing these approaches is necessary to establish direct mechanistic links between insulin treatment, PKM2 activation, and placental metabolic shifts.

The significantly higher PKM2 and pAMPK expression in GDM-I placentas may reflect a stronger metabolic response to insulin-driven glucose uptake, while the more restrained activation in GDM-D placentas suggests dietary management maintains a more moderate metabolic state. While an increase in PKM2 in GDM-I placentas is correlated with insulin treatment, it is important to distinguish whether insulin therapy is directly responsible for this change or if it is an adaptive response to underlying metabolic stress. Insulin increases glucose uptake via GLUT transporters in various tissues, including the placenta, which could lead to a shift towards glycolysis and consequently the upregulation of PKM2 [34]. However, this does not necessarily mean insulin is the direct cause of PKM2 induction. Other factors, such as increased glucose flux and metabolic stress associated with insulin resistance, may contribute to PKM2 upregulation independent of insulin therapy [4]. Additionally, inflammatory cytokines known to be elevated in GDM, including TNF-α and IL-6 [31], have been implicated in PKM2 regulation and may further drive its activation [35,36]. To establish causation, future studies should evaluate PKM2 expression in insulin-resistant placentas from patients not receiving exogenous insulin therapy, as well as explore potential direct regulatory effects of insulin on PKM2 at the transcriptional and post-translational levels. The implications of these differences warrant further study to determine whether treatment type affects placental function and fetal outcomes differently.

PKM2 plays a dual role in placental metabolism, balancing glycolytic energy production and nuclear signaling. In physiological contexts, PKM2 activation plays a crucial role in supporting cellular energy production under conditions of metabolic stress. In the placenta, a tissue with high energetic demands, increased glycolysis facilitated by PKM2 activation may serve as an adaptive mechanism to maintain ATP production in response to the altered metabolic environment of GDM [37]. Enhanced glycolytic capacity may help placental cells compensate for insulin resistance by providing an alternative ATP source when oxidative phosphorylation is impaired [38]. However, while glycolytic adaptation may be beneficial in sustaining placental function, excessive reliance on glycolysis can also lead to metabolic inefficiencies. The accumulation of glycolytic intermediates may promote the formation of byproducts such as lactate, which could alter placental microenvironmental pH and impact nutrient transport to the fetus [39]. Additionally, PKM2’s ability to translocate to the nucleus and regulate gene expression suggests that its upregulation in GDM may have downstream effects beyond energy metabolism, potentially influencing trophoblast proliferation and inflammatory signaling [2]. Therefore, while PKM2 activation may initially help sustain energy homeostasis in GDM placentas, prolonged or excessive activation may contribute to metabolic dysfunction. Our findings demonstrate that GDM placentas favor the active (tetrameric) form of PKM2, suggesting a metabolic shift to sustain glycolysis under hyperglycemic conditions. Insulin resistance, a hallmark of GDM, leads to compensatory hyperinsulinemia, which promotes glycolytic metabolism and alters the placental energy balance [4]. In response to insulin resistance, placental cells may shift towards an increased reliance on glycolysis, leading to PKM2 upregulation as a means to sustain ATP production. This mechanism is supported by findings in cancer metabolism, where PKM2 is upregulated in response to metabolic stress to facilitate glycolytic adaptation [40].

Notably, PKM2 expression was further elevated in GDM-I placentas, with localization predominantly in the syncytiotrophoblast layer, which is responsible for nutrient and gas exchange. Insulin treatment may contribute to PKM2 activation by enhancing glucose uptake and promoting glycolytic metabolism. High glucose conditions have been shown to stabilize the tetrameric, active form of PKM2, favoring glycolysis over oxidative phosphorylation [41]. Given that GDM-I patients require insulin therapy due to more pronounced insulin resistance, it is likely that both endogenous insulin resistance and exogenous insulin treatment contribute to PKM2 activation. The higher PKM2 expression in GDM-I placentas may reflect an increased reliance on glycolysis due to insulin-stimulated glucose availability [1]. Conversely, GDM-D placentas showed a more moderate increase in PKM2 expression, suggesting dietary intervention maintains a more balanced metabolic state. These findings align with previous reports that metabolic stress in pregnancy induces PKM2-dependent adaptations [13,14].

In GDM-I placentas, we observed a decrease in phosphorylated PKM2 (pPKM2), suggesting a reduction in the inactive, nuclear-localized form. This shift may facilitate glycolysis over nuclear functions, highlighting a potential metabolic adaptation of placental cells to meet heightened energy demands during GDM. The variation in PKM2 activation between GDM-I and GDM-D placentas may result from several factors, including differences in insulin sensitivity, glucose availability, and metabolic adaptation. Insulin treatment directly enhances glucose uptake in placental cells, leading to an increased glycolytic flux and potentially higher PKM2 expression in GDM-I placentas compared to GDM-D. This metabolic shift aligns with the Warburg effect, a phenomenon where cells preferentially utilize glycolysis over oxidative phosphorylation despite oxygen availability [42]. Additionally, PKM2 activation may be modulated by the level of insulin resistance, which differs between GDM-I and GDM-D patients. Insulin-treated patients generally exhibit greater insulin resistance, necessitating exogenous insulin administration, which may further drive metabolic adaptations. Furthermore, hormonal variations and cytokine levels may contribute to PKM2 regulation, given their known effects on metabolic enzyme expression Therefore, the differences in PKM2 activation between the GDM-I and GDM-D placentas may stem from both treatment-induced metabolic shifts and underlying insulin resistance severity.

AMPK is a master regulator of energy homeostasis, activated under low-energy conditions to restore ATP levels [19]. Its activation in response to elevated AMP/ATP ratios promotes catabolic pathways and enhances glucose uptake, making it an attractive target for metabolic disease intervention, including diabetes [20]. The significant increase in pAMPK levels, particularly in GDM-I placentas, supports its role as a metabolic sensor in response to the energy stress induced by GDM. This activation of AMPK may represent an effort by the placenta to counteract GDM-induced insulin resistance, promoting glucose uptake and utilization to support fetal growth [6,21]. Interestingly, the correlation between increased AMPK and PKM2 in GDM-I placentas aligns with findings in cancer studies, where AMPK activation has been shown to support metabolic adaptations in cells with high proliferative demands [31]. In the context of the placenta, enhanced AMPK activity may help compensate for GDM-induced metabolic dysregulation by maintaining energy production through glycolysis and other catabolic pathways. There is a differential activation of AMPK in GDM-I versus GDM-D placentas, as AMPK activation has been shown to enhance glycolysis and PKM2 activity in metabolically stressed cells [31]. However, the chronic activation of AMPK could also contribute to placental oxidative stress, potentially exacerbating GDM-related placental dysfunction.

The mTOR pathway, which regulates cell growth and metabolism in response to nutrient and energy availability, was also significantly altered in GDM placentas [12]. Increased mTOR activation in GDM-I placentas, as evidenced by elevated phosphorylated mTOR (pmTOR) levels, suggests that insulin treatment may upregulate nutrient-sensing pathways. mTOR signaling is known to be influenced by insulin and nutrient status, which can enhance cellular proliferation and growth by increasing protein synthesis and energy expenditure [12]. This finding is significant given that excessive mTOR activation has been associated with increased placental growth and fetal overgrowth (macrosomia) [43]. Insulin stimulates mTOR activity via the Akt pathway, promoting protein synthesis and cell proliferation. In placental tissue, excessive mTOR activation may contribute to trophoblast hyperplasia, increasing placental size and nutrient transfer to the fetus, thereby predisposing the offspring to macrosomia [44]. The absence of increased pmTOR in GDM-D placentas highlights a differential regulatory effect depending on the treatment modality, possibly due to lower insulin exposure and the absence of exogenous insulin stimulation, which could have implications for fetal growth outcomes in GDM. This suggests that dietary management may prevent excessive placental overgrowth by limiting hyperinsulinemia and subsequent mTOR upregulation.

Interestingly, the differential effects on downstream mTOR targets, such as increased active p70 in GDM-I but decreased active p70 in GDM-D placentas, suggest distinct metabolic adjustments depending on treatment type. This divergence may reflect varying nutrient availability and metabolic demand within the placental tissue, potentially influencing fetal nutrient supply. The unique expression patterns of mTOR-related proteins in response to insulin versus dietary interventions underscore the importance of tailored therapeutic approaches in GDM.

The observed alterations in PKM2, AMPK, and mTOR pathways in GDM placentas underscore the potential of these pathways as therapeutic targets. The interaction among PKM2, AMPK, and mTOR is central to the metabolic regulation of the placenta in GDM. PKM2, a key glycolytic enzyme, facilitates ATP production under conditions of metabolic stress, thereby supporting placental energy demands [37]. However, its upregulation is often accompanied by enhanced mTOR signaling, which regulates nutrient sensing and trophoblast proliferation, potentially leading to excessive fetal growth [12]. AMPK, acting as an energy sensor, serves as a counter-regulatory mechanism by suppressing mTOR activity when ATP levels are low, thereby promoting cellular energy conservation [4]. Our findings indicate that in insulin-treated GDM placentas, mTOR activation is more pronounced, whereas AMPK upregulation is more evident in diet-managed pregnancies, suggesting a fundamental difference in metabolic adaptation between these treatments. Understanding the balance between these pathways may be critical for developing targeted therapies that optimize placental metabolism while preventing complications such as macrosomia or fetal growth restriction. Further studies should investigate whether the pharmacological modulation of these pathways could provide therapeutic benefits in GDM. PKM2 modulation, for instance, may be a promising target to optimize glycolysis and energy balance in the GDM placenta, ensuring adequate energy supply to support fetal development. Pharmacological agents that stabilize the tetrameric form of PKM2, thereby promoting glycolytic flux, could potentially counteract the energy deficit observed in GDM placentas, although such interventions would require a careful consideration of PKM2’s pleiotropic effects on cell proliferation and gene expression [45].

Moreover, AMPK activators like metformin have shown efficacy in improving insulin sensitivity and reducing hyperglycemia in diabetes, including GDM, and may also benefit placental energy metabolism by enhancing glycolytic flux and glucose uptake. However, the impact of AMPK activation on placental oxidative stress and fetal development remains a critical consideration, warranting further research to ensure safety and efficacy in GDM treatment [19].

Additionally, modulating the mTOR pathway could serve as a targeted approach to regulate placental growth and function in GDM. As mTOR signaling influences both cell proliferation and nutrient transport, its selective inhibition in cases of excessive activation, as observed in the GDM-I placentas, could potentially mitigate hyperplasia and abnormal nutrient transport. However, given the complexity of the mTOR pathway and its involvement in various cellular processes, careful dose-dependent management would be crucial to avoid adverse effects on fetal growth and development.

One limitation of this study is the relatively modest sample size (n = 10 per group), which may restrict the generalizability of our findings. While our study provides valuable insights into the differential metabolic adaptations in insulin-treated (GDM-I) and diet-managed (GDM-D) pregnancies, a larger cohort with more diverse demographic and clinical characteristics would help validate these findings. Expanding the sample size would allow for a more comprehensive evaluation of potential confounders, including maternal BMI, pre-pregnancy metabolic status, and genetic predisposition to insulin resistance. Additionally, more extensive studies could facilitate subgroup analyses to determine whether treatment effects vary by gestational age or the severity of GDM. Future research should aim to replicate our findings in multicenter studies encompassing broader patient populations, ensuring that conclusions drawn from metabolic profiling in GDM placentas are robust and widely applicable [4].

Future research should delineate the precise molecular mechanisms by which GDM alters the placenta’s PKM2, AMPK, and mTOR pathways. The differential activation of PKM2 and mTOR in GDM placentas suggests that insulin therapy may amplify metabolic adaptations beyond those observed with dietary interventions. Given the association between mTOR signaling and fetal overgrowth, further research is needed to determine whether modifying treatment strategies could mitigate macrosomia risk in insulin-treated GDM pregnancies. Additionally, studies should evaluate whether alternative therapies, such as combination approaches (diet + low-dose insulin or metformin), may provide metabolic benefits while minimizing excessive mTOR activation. Longitudinal studies tracking the metabolic profile of GDM placentas from early to late gestation could provide insights into the progression of these molecular changes.

Additionally, understanding how these pathways interact with other placental factors, such as oxidative stress markers and inflammatory cytokines, could reveal more comprehensive intervention strategies for GDM management. Given the role of insulin treatment in exacerbating specific molecular alterations in GDM placentas, there is a need to evaluate alternative therapies or combination treatments that could minimize adverse effects while optimizing glucose regulation. Given the role of insulin treatment in exacerbating specific molecular alterations in GDM placentas, there is a need to evaluate alternative therapies or combination treatments that could minimize adverse effects while optimizing glucose regulation. A key aspect that remains unexplored is how PKM2, AMPK, and mTOR expression and activation change throughout gestation in GDM pregnancies. Our study provides a cross-sectional analysis of metabolic adaptations in term placentas; however, longitudinal studies tracking these pathways from early to late gestation would offer critical insights into the progression of metabolic dysregulation in GDM. Monitoring mTOR activation over time could help determine whether its upregulation in GDM-I placentas is an early compensatory response to insulin resistance or a progressive adaptation that exacerbates fetal overgrowth [12]. Similarly, assessing PKM2 and AMPK activity across gestation could clarify whether increased glycolytic reliance is a transient response to hyperglycemia or a sustained metabolic shift.

Longitudinal studies incorporating maternal metabolic profiling, fetal growth parameters, and placental function assessments would provide a more comprehensive understanding of the interplay between maternal glycemic control and placental metabolism. Such studies could inform targeted interventions to optimize metabolic balance throughout pregnancy to reduce adverse outcomes associated with GDM. Diet and lifestyle modifications, potentially in conjunction with pharmacological agents targeting specific metabolic pathways, might offer a more balanced approach to managing GDM. These findings underscore the potential for personalized treatment strategies in GDM based on the metabolic profiling of the placenta. Given the differential activation of metabolic pathways in GDM-I and GDM-D placentas, individualized treatment planning could optimize metabolic regulation while minimizing adverse effects such as excessive fetal growth. For instance, placental mTOR activation may be a biomarker to predict fetal overgrowth risk in insulin-treated pregnancies, guiding adjustments in insulin dosage or introducing adjunct therapies such as metformin [12]. Similarly, assessing PKM2 activation could inform whether dietary management is sufficient or if additional pharmacologic support is needed to maintain placental energy homeostasis. By integrating metabolic profiling into clinical decision making, treatment strategies could be tailored to individual patients, ensuring optimal maternal glucose control and favorable fetal metabolic outcomes. Future research should address current limitations, including sample size constraints, the need for mechanistic in vitro studies, and longitudinal investigations of metabolic regulation throughout pregnancy. Understanding how PKM2, AMPK, and mTOR pathways evolve over gestation will be critical in refining therapeutic strategies to optimize maternal and fetal health outcomes in GDM pregnancies.

## Figures and Tables

**Table 1:** Shows the demographic information of the collected samples.

	Control	GDM-D	GDM-I
Maternal Age	34 ± 1.6	33 ± 1.5	35 ± 1.4
Gestational Age (weeks)	38 ± 0.09	38 ± 0.3	39 ± 0.8
Fetal Weight (g)	3498 ± 59	3245 ± 151	3453 ± 217
% C-section/Vaginal	90%/10%		

## Data Availability

The original contributions presented in the study are included in the article; further inquiries can be directed to the corresponding author.
